# Anti-SARS-CoV-2 Receptor-Binding Domain Total Antibodies Response in Seropositive and Seronegative Healthcare Workers Undergoing COVID-19 mRNA BNT162b2 Vaccination

**DOI:** 10.3390/diagnostics11050832

**Published:** 2021-05-04

**Authors:** Gian Luca Salvagno, Brandon M. Henry, Giovanni di Piazza, Laura Pighi, Simone De Nitto, Damiano Bragantini, Gian Luca Gianfilippi, Giuseppe Lippi

**Affiliations:** 1 Section of Clinical Biochemistry, University of Verona, 37126 Verona, Italy; gianluca.salvagno@univr.it (G.L.S.); laura.pighi@studenti.univr.it (L.P.); simone.denitto@studenti.univr.it (S.D.N.); 2 Service of Laboratory Medicine, Pederzoli Hospital, 37019 Peschiera del Garda, Italy; 3 Cincinnati Children’s Hospital Medical Center, The Heart Institute, Cincinnati, OH 45229, USA; brandon.henry@cchmc.org; 4 Medical Direction, Pederzoli Hospital, 37019 Peschiera del Garda, Italy; gdipiazza@ospedalepederzoli.it (G.d.P.); ggianfilippi@ospedalepederzoli.it (G.L.G.); 5 Infectious Diseases Unit, Pederzoli Hospital, 37019 Peschiera del Garda, Italy; dbragantini@ospedalepederzoli.it

**Keywords:** COVID-19, SARS-CoV-2, vaccine, immune response, antibodies

## Abstract

Background: This study monitored total anti-SARS-CoV-2 (severe acute respiratory syndrome coronavirus 2) RBD (receptor-binding domain) antibodies levels in a large population of healthcare workers undergoing mRNA COVID-19 vaccination. Methods. The study population consisted of employees of Pederzoli Hospital of Peschiera del Garda (Verona, Italy), who underwent voluntary vaccination with two doses of COVID-19 mRNA BNT162b2 (Comirnaty; Pfizer Inc). Venous blood was drawn immediately before the first vaccine dose, as well as 21 days (immediately before second vaccine dose) and 50 days afterwards. Humoral response was assessed with Roche Elecsys Anti-SARS-CoV-2 S total antibodies, on Roche Cobas 6000 (Roche Diagnostics). Results: The final study population consisted of 925 subjects (mean age, 44 ± 13 years; 457 women), 206 (22.3%) anti-SARS-CoV-2 baseline seropositive. The increase of total anti-SARS-CoV-2 RBD antibodies levels 21 days after the first vaccine dose was ~3 orders of magnitude higher in seropositive than in seronegative individuals (11782 vs. 42 U/mL; *p* < 0.001). Total anti-SARS-CoV-2 RBD antibodies levels further increased by over 30-fold after the second vaccine dose in baseline seronegative subjects, while such increase was only ~1.3-fold in baseline seropositive subjects. In multivariate analysis, total anti-SARS-CoV-2 RBD antibodies level was inversely associated with age after both vaccine doses and male sex after the second vaccine dose in baseline seronegative subjects, while baseline antibodies value significantly predicted immune response after both vaccine doses in baseline seropositive recipients. Conclusion: Significant difference exists in post-mRNA COVID-19 vaccine immune response in baseline seronegative and seropositive subjects, which seems dependent on age and sex in seronegative subjects, as well as on baseline anti-SARS-CoV-2 antibodies level in seropositive patients.

## 1. Introduction

One and two decades after the two last coronavirus outbreaks sustained by severe acute respiratory syndrome coronavirus (SARS-CoV) and Middle-East respiratory syndrome coronavirus (MERS-CoV), respectively, a new coronavirus disease has emerged, reaching pandemic proportions [1]. Coronavirus disease 2019 (COVID-19), caused by severe acute respiratory syndrome coronavirus 2 (SARS-CoV-2), generates a wide spectrum of clinical manifestations, from asymptomatic infection to severe lower respiratory tract involvement (with interstitial pneumonia), then progressing toward severe systemic disease and development of multiple organ failure in patients with severe illness, up to death [2]. Owing to the nearly unstoppable worldwide diffusion of SARS-CoV-2, several preventative and containing measures have been adopted. Besides physical interventions, such as social distancing, widespread use of face masks, hand hygiene and timely isolation of infected and infectious subjects, universal vaccination is now regarded as the most effective strategy to limit the clinical, societal and economic consequences of COVID-19 around the world [3].

Vaccines mainly act by simulating a natural infection, and thereby promoting development of a humoral and cellular immune response aimed at defending the host against a specific pathogen. As specifically concerns COVID-19, unprecedented efforts have been underway to develop efficient vaccine formulations primarily aimed at reducing the risk of developing aggressive forms of disease, and hence preventing healthcare system collapse, as well as for limiting the encumbrance of asymptomatic infections, which may still actively contribute to sustain viral circulation within the community [4]. Several strategies are being pursued, thus encompassing vaccines based on inactivated virus, viral proteins (e.g., spike protein), as well as DNA- and mRNA-based vaccines [5]. 

The last generation of lipid-based mRNA-lipid nanoparticles vaccines (mRNA-LNPs) was found to be especially convenient, since they conjugate many technical, biological and clinical advantages [6]. The major technical advantages of these mRNA-LNPs are represented by their ability to closely reproduce natural viral infection without delivering viral particles (mRNA penetrates the host cells and is translated into antigen viral proteins mounted at cell surface or released in the surrounding environment), the potentially lower immunogenicity and cytotoxicity of nanoparticles, the capability to deliver multimeric antigens which may hence allow the rapid reengineering of the formulation with inclusion of new polymorphisms [7,8]. With respect to the biological aspects, the currently used mRNA-LNPs contain genetic material encoding a recombinant form of SARS-CoV-2 spike protein with its receptor-binding domain (RBD), and are hence effective to stimulate B cells to generate neutralizing antibodies directed against the spike protein, thus reducing binding effectiveness with receptors at the host cell surface (especially with angiotensin converting enzyme 2; ACE2), and enhancing virus inactivation and clearance. A sustained generation of anti-SARS-CoV-2 T cells is also likely elicited (especially CD4+ and CD8+ cells), and would work to eliminate infected cells [9]. These two convergent pathways synergistically contribute to mitigate the clinical impact of SARS-CoV-2, as attested by recent evidence reviewed by Abdool Karim and de Oliveira [10], showing that mRNA-LNPs may display 94–95% efficiency in preventing SARS-CoV-2 infection and 90–100% efficacy in averting severe COVID-19 illness. Nonetheless, concerns have been expressed that the immune response after administration of these mRNA-LNPs may be characterized by high inter-individual variation, with some people developing higher titers of neutralizing antibodies compared to others who may only have a “mild” and thus less efficient response [11,12,13]. Therefore, this retrospective observational study was aimed to monitor the anti-SARS-CoV-2 RBD total antibodies response in a large population of healthcare workers, both SARS-CoV-2 seropositive and seronegative, undergoing voluntary mRNA vaccine administration.

## 2. Materials and Methods

The study population consisted of the entire sanitary and administrative staff of the Pederzoli Hospital of Peschiera del Garda (Verona, Italy), who underwent voluntary vaccination with COVID-19 mRNA-LNP BNT162b2 (Comirnaty; Pfizer Inc, NY, USA). The first 30 μg vaccine dose was administered between January 4 and 15, 2021, followed by a second 30 μg vaccine dose 21 days exactly after the first dose. Both vaccine doses were prepared strictly following manufacturer’s instruction and administered to all study participants within 30 min from resuspension. No subjects had taken immunosuppressive drugs immediately before vaccination. Venous blood was drawn in the morning by straight needle venipuncture into evacuated blood tubes containing gel and clot activator (Greiner Bio-One, Kremsmünster, Austria) 15 min before administration of the first vaccine dose, as well as 21 (immediately before 2nd dose vaccination) and 50 days afterwards. Blood samples were transported to the local core laboratory, where they were separated by centrifugation for 15 min at 1500× *g* at room temperature. Serum was separated from the underlying cellular pellet, divided in 2 identical aliquots of ~1.5 mL and stored at −70°C until measurement. Therefore, all subjects were prospectively enrolled for vaccination, according to national guidelines, and then samples were retrospectively analyzed. The paired aliquots collected at different time points from each participant were concurrently thawed at the end of the study period, centrifuged and analyzed with the novel Roche Elecsys Anti-SARS-CoV-2 S immunoassay on a Roche Cobas 6000 (Roche Diagnostics, Basel, Switzerland). This one-step double antigen sandwich assay has been developed for quantitative assessment of total anti-SARS-CoV-2 RBD antibodies in human serum and plasma specimens. Briefly, the patient sample is incubated with a mix of biotinylated and ruthenylated SARS-CoV-2 RBD recombinant antigen. Double antigen sandwich immune complexes are eventually formed when the corresponding antibodies are present. After addition of streptavidin-coated microparticles, double antigen sandwich immune complexes bind to the solid phase through interaction of biotin and streptavidin. The reagent mix is then transferred to the measuring cell, where microparticles are magnetically captured onto the electrode surface. Unbound material is removed and electrochemiluminescence is applied and measured with a photomultiplier. The signal yield is proportional to total anti-SARS-CoV-2 RBD antibodies level present in the test sample. According to manufacturer’s declaration, this test displays 92% (95% CI, 64–100%) positive agreement with a virus pseudo-neutralization assay and 100% diagnostic specificity and 89% diagnostic sensitivity for detecting SARS-CoV-2 infection 14 days after symptoms onset. The limit of blank (LoB) and limit of detection (LoD) are 0.30 U/mL and 0.40 U/mL, respectively, the linearity is between 0.40–250 U/mL (extensible to 2500 U/mL with 1:10 sample dilution), and the total imprecision is between 1.4–2.4%. Test results <0.8 U/mL are classified as non-reactive, while those ≥0.8 U/mL are classified as reactive. A recent clinical evaluation of this novel immunoassay found excellent performance, with 97.9% and 100% positive agreement with molecular testing >14 days and >21 days after symptoms onset, respectively, combined with 99.9% negative agreement [14]. 

All subjects participating to this retrospective observational study gave two separate written informed consents for both receiving vaccination and being included in the serological monitoring survey. This retrospective observational study was conducted in accordance with the Declaration of Helsinki and the protocol cleared by the Ethics Committee of the Provinces of Verona and Rovigo (3246CESC).

### Statistical Analysis

The results of total anti-SARS-CoV-2 RBD antibodies testing were presented as median and interquartile range (IQR), and as ratio with baseline total anti-SARS-CoV-2 antibodies level (i.e., [time point value/baseline value and/or limit of detection]). Differences between groups were assessed with Mann–Whitney U test and chi-square statistics, when appropriate. Univariate relationships between antibody levels and other variables (e.g., age, sex, baseline total anti-SARS-CoV-2 RBD antibody level) were assessed using Spearman’s correlation. Multivariable linear regression analyses were then used to assess these correlations for each time point (day 21 and day 50) and group (seropositive and seronegative). The mean difference (MD) with 95% confidence interval (95% CI) was calculated to quantify the difference of total anti-SARS-CoV-2 RBD antibodies levels between groups. Statistical analysis was conducted with Analyse-it (Analyse-it Software Ltd, Leeds, UK) and MetaXL, software Version 5.3 (EpiGear International Pty Ltd., Sunrise Beach, Australia). 

## 3. Results

**Figure 1:**
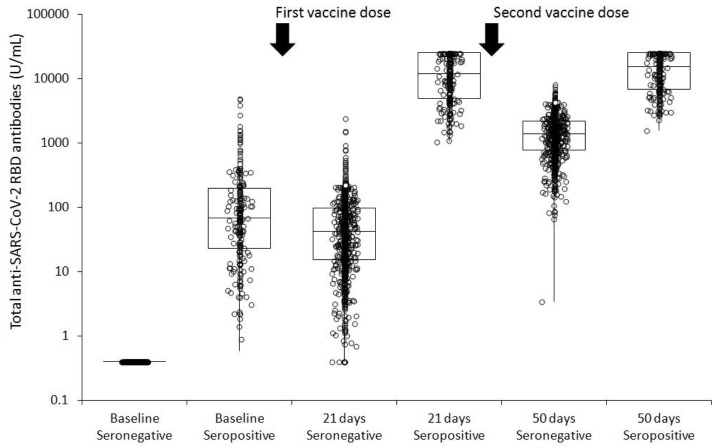
Total anti-SARS-CoV-2 RBD antibodies in seronegative (<0.8 U/L) or seropositive (≥0.8 U/L) subjects at baseline and after receiving a complete cycle (i.e., two doses) of Pfizer COVID-19 mRNA BNT162b2 vaccine.

The initial study population consisted of 1003 employees of the Pederzoli Hospital of Peschiera del Garda, who voluntarily agreed to undergo vaccination with Pfizer COVID-19 mRNA Vaccine Comirnaty. A total number of 78 subjects were lost during follow-up sampling (7 at 21 days and 71 at 50 days, respectively), such that the final study population consisted of 925 subjects (mean age, 44 ± 13 years; 457 (49.4%) women) who completed the two-dose vaccine cycle and had serum samples drawn at all the three time points. Two hundred and six (22.3%) subjects had measurable total anti-SARS-CoV-2 RBD antibodies level (i.e., ≥0.8 U/mL) before vaccination, and were hence classified as baseline seropositive. The age (43 ± 13 vs. 44 ± 13 years; *p* = 0.206) and sex (70% vs. 64% females; *p* = 0.093) of baseline seropositive individual values did not differ from those of baseline seronegative subjects. The humoral immune response in both the seronegative and seropositive cohorts after the complete mRNA COVID-19 vaccination cycle is shown in Figure 1. 

The absolute increase of antibody level 21 days after the first vaccine dose was nearly 3 orders of magnitude higher in seropositive than in seronegative individuals (11782 vs. 42 U/mL; *p* < 0.001) (Table 1). 

While antibodies levels further increased by over 30 folds 1 month after the second vaccine dose in baseline seronegative subjects, the increase in baseline seropositive subjects was only ~1.30-fold (Table 1). Nonetheless, 1 month after the second vaccine dose, baseline seropositive subjects had total anti-SARS-CoV-2 RBD antibodies levels that were 11-fold higher than baseline seronegative subjects (15142 vs. 1364 U/mL; *p* < 0.001). The rate of subjects reaching seropositive status after receiving the first vaccine dose was 98.7% and 100% in baseline seronegative and seropositive recipients, respectively, increasing to 100% in both cohorts 1 month after the second vaccine dose. A highly significant correlation could be found between the ratios of increase from the individual baseline antibodies level observed after the first (21 days) and second (50 days) vaccine doses in both baseline seronegative (*r* = 0.68; 0.63 to 0.71; *p* < 0.001) and baseline seropositive (*r* = 0.95; 95% CI, 0.94 to 0.96; *p* < 0.001) recipients.

The Spearman’s correlation between the magnitude of increase in total anti-SARS-Cov-2 antibodies and age, sex, and baseline antibodies levels in baseline seronegative and seropositive subjects is shown in Table 2. In univariate analysis, total anti-SARS-CoV-2 RBD antibodies response to the first and second vaccine doses was significantly associated with both age and sex in the baseline seronegative cohort, while it was only associated with baseline antibody value in those seropositive at baseline (Table 2).

In multivariate analysis, total anti-SARS-CoV-2 RBD antibodies response remained significantly associated with age after both vaccine doses and with sex after the second vaccine dose in baseline seronegative subjects, while the baseline total anti-SARS-CoV-2 RBD antibodies value remained significant predictor of response after both vaccine doses in the baseline seropositive cohort (Table 3).

**Figure 2:**
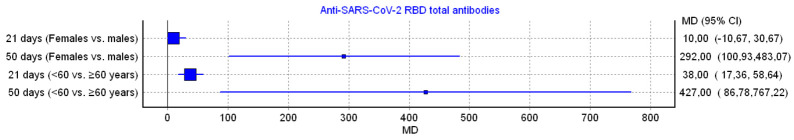
Mean difference (MD) of total anti-SARS-CoV-2 RBD antibodies in seronegative subjects receiving a complete cycle (i.e., two doses) of Pfizer COVID-19 mRNA BNT162b2 vaccine as calculated after 21 and 50 days from the first dose.

The MD of total anti-SARS-CoV-2 RBD antibody levels among different cohorts is summarized in Figure 2. After the second vaccine dose, women (*n* = 457; 63.6%) had 292 U/mL higher levels than men (+1.20-fold), while subjects younger than 60 years (*n* = 644; 89.6%) had 38 U/mL (+1.84-fold) and 427 U/mL (+1.33-fold) higher antibody levels after the first and second vaccine dose, respectively, compared to older individuals (Figure 2).

## 4. Discussion

It seems now almost indisputable that universal COVID-19 vaccination will be the keystone in all strategies aimed at stopping or limiting the worldwide circulation of SARS-CoV-2. Nonetheless, although the efficacy of most currently licensed vaccines appears considerably high, especially at reducing the risk of clinical deterioration [15], a considerable inter-individual heterogeneity in post-vaccine immune response is being increasingly noted in some specific populations, especially in the elderly [16] and in immunosuppressed patients [17,18]. Unfortunately, low vaccine responders mostly include categories of frail patients, who already have a magnified risk of unfavorable disease progression if contracting SARS-CoV-2 [19]. To this end, monitoring post-vaccine immune response in the population should be considered highly advisable, as also recently endorsed by the International Federation of Clinical Chemistry and Laboratory Medicine (IFCC) [20].

Overall, the identification of three important predictors (age, sex, baseline serostatus) of post-COVID-19 mRNA BNT162b2 vaccine humoral response in our population of healthcare workers may have some substantial implications and consequences for vaccine plans. Unlike the recent study of Dörschug et al. [21], who used a spike protein-based IgG serological immunoassay for monitoring humoral response to COVID-19 mRNA BNT162b2 vaccine and failed to find significant differences between sexes, we found that women had a significantly higher response (between 1.15–1.20-fold higher compared to men) of total anti-SARS-CoV-2 RBD antibodies, especially after the second vaccine dose. This agrees with recent data published by Terpos et al. [22], who also found that the anti-Spike-RBD IgGs response was more sustained in female than in male octogenarians after vaccination with Pfizer BNT162b2 mRNA vaccine. In keeping with previous reports [23,24,25,26], we also observed a gradual reduction of total anti-SARS-CoV-2 RBD antibodies level in older individuals (i.e., aged 60 years or older). In particular, a significant difference by age (≤60 years vs. >60 years) could be noted after the first mRNA vaccine dose (~1.3-fold), which was magnified after the second dose (~1.9-fold). Interestingly, the highly significant correlation observed in the immune response observed after the first and second vaccine doses in both baseline seronegative and baseline seropositive recipients suggests that the final level of total anti-SARS-CoV-2 RBD antibodies attainable after the second vaccine dose could be reliably predicted by the first dose response, especially in baseline seropositive recipients.

The lower total anti-SARS-CoV-2 RBD antibodies response found in males and older baseline seronegative subjects would suggest that this specific population may have less efficient protection against infection and/or an even higher risk of developing more aggressive forms of COVID-19, such that delaying, or even abolishing, the second vaccine dose seems highly unadvisable [27]. Other than further safeguarding these populations from being infected by SARS-CoV-2 by encouraging public health preventative measures, higher and/or more frequent mRNA vaccine doses should be considered for boosting the immunogenicity in subjects with predictably lower response after the standard 2-dose mRNA vaccine cycle, as also recently underpinned by Van Praet and colleagues [28]. This hypothesis is already under consideration by some pharmaceutical companies. Pfizer has already started to test the efficacy of a third booster in people aged 65 to 85 years, who have received their first two doses of COVID-19 mRNA BNT162b2 in the phase III trial [29]. 

Assuring a sustained and durable protection against SARS-CoV-2 in the community by vaccination is the most important tool for containing the dramatic clinical and societal effects of the ongoing SARS-CoV-2 pandemic in the face of the emergence of novel strains and new outbreaks. To this end, the identification of subjects with low/modest post-vaccine immune response will be vital for limiting the potential unfavorable impact of emerging SARS-CoV-2 variants (especially those bearing the so-called “immune escape mutations”), ensuring adequate personal and community immune protection and limiting viral circulation, thereby reducing the risk that novel and even more dangerous mutations will accumulate [30,31,32]. Moreover, recent evidence of a sustained IgA response after mRNA-LNPs vaccination [33], combined with clinical observations of decreased viral load in the limited number of reported cases after inoculation [34], suggests that these vaccines can reduce viral replication, which itself would result in decreased likelihood of new mutation generation in the case of asymptomatic/symptomatic SARS-CoV-2 infection of a vaccinated individual.

The influence of baseline anti-SARS-CoV-2 antibody status on post-vaccine response of seropositive subjects (i.e., those with previous asymptomatic or symptomatic infection) deserves special focus. We observed enormously boosted total anti-SARS-CoV-2 RBD antibodies response in baseline seropositive people, which may have some notable clinical implication. Several studies have now reported that the level of anti-SARS-CoV-2 antibodies reached after vaccination is dependent on the seronegative/seropositive status at time of vaccination [35,36,37,38,39,40]. In particular, Mueller also used the Roche Elecsys Anti-SARS-CoV-2 S immunoassay to monitor response to BNT162b2 COVID-19 vaccine and found that antibodies became positive in all samples 2 weeks after the first administration, with serum concentrations then constantly increasing for the following 4–5 weeks [41]. Although our findings are indeed in substantial agreement with these previous observations, we provide further evidence that the baseline antibody status is a very strong predictor of post-vaccine total anti-SARS-CoV-2 RBD antibodies response, with high correlation after the first and second vaccine doses. Our data clearly show that the second vaccine dose only produced a marginal gain of antibodies titer (i.e., around 30%) after the first dose in our population of baseline seropositive subjects. Considering the very high anti-SARS-CoV-2 RBD total antibodies levels seen after the first vaccine dose, the second dose is unlikely to significantly enhance protection against re-infections, even with different strains, and/or the risk of developing severe illness, at least within a 6- to 12-month window after vaccination when IgG titers will likely remain adequate in most. With vaccines supply remaining limited all around the world [42], the European Center for Disease Control and Prevention (ECDC) states that consideration should be given to vaccinate specific populations which may have disproportionate risk of exposure or disease aggravation [43]. In keeping with this suggestion, allocation of vaccine doses for those who are at high risk of severe disease should be a universal priority [44,45]. 

Evidence is accumulating that people who have been previously infected with SARS-CoV-2, with either symptomatic or asymptomatic disease, may be predisposed to stronger reactions to vaccination, thus carrying a greater risk of developing side effects and/or adverse reactions [38]. This risk has been clearly acknowledged by the US Centers for Disease Control and Prevention in its ad interim clinical recommendations for COVID-19 vaccination [46], whereby it is now stated that people with a recent SARS-CoV-2 infection may choose to temporarily delay vaccination. Finally, the considerably high anti-SARS-CoV-2 baseline positivity observed in our study (i.e., 22.3%) is not surprising, since an almost identical figure (i.e., 22.9%) has been reported in a seroprevalence survey recently carried out in the General Hospital of Brescia [47], which is just at 50 km distance from Peschiera del Garda.

## 5. Conclusions

The results of this large retrospective observational study aimed at monitoring total anti-SARS-CoV-2 RBD antibodies response after Pfizer mRNA COVID-19 vaccination reveal that significant difference exists between seronegative and seropositive subjects, and that such response may be dependent on age and sex in seronegative subjects, as well as on baseline anti-SARS-CoV-2 antibodies level in seropositive patients. Future studies should be planned to establish whether additional demographical variables not included in our analysis, such as the presence of comorbidities, ethnicity, body mass index, and physical activity, may influence humoral response to mRNA-LNPs vaccination.

## Figures and Tables

**Table 1:** Total anti-SARS-CoV-2 RBD antibodies levels (median and interquartile range) in seronegative (<0.8 U/L) or seropositive (≥0.8 U/L) subjects at baseline and after receiving a complete cycle (i.e., two doses) of Pfizer COVID-19 mRNA BNT162b2 vaccine.

Antibody Status	*n*	Age (Years)	Sex (Females)	Baseline	21 Days	50 Days
Seronegative						
- Level (u/mL)	719	44 ± 13	457/719 (64%)	<0.8	42 (15–98)	1364 (761–2174)
- ≥0.8 u/mL (%)				0/709 (0%)	710/719 (98.7%)	719/719 (100%)
Seropositive						
- Level (u/mL)	206	43 ± 13	144/206 (70%)	68 (23–194)	11782 (4848–25,000)	15142 (6824–25,000)
- ≥0.8 u/mL (%)				206/206 (100%)	206/206 (100%)	206/206 (100%)

**Table 2:** Spearman’s correlation coefficient (and 95% CI) between the ratio of increase from individual baseline total anti-SARS-CoV-2 RBD antibodies level and age, sex or baseline antibodies value in baseline seronegative (<0.8 U/L) and baseline seropositive (≥0.8 U/L) subjects receiving a complete cycle (i.e., two doses) of Pfizer COVID-19 mRNA BNT162b2 vaccine.

Antibody Status	Age	Sex (Males vs. Females)	Baseline Value
Seronegative			
- 21 days	−0.33 (95%CI, −0.39 to −0.26; *p* < 0.001)	−0.08 (95%CI, −0.15 to −0.01; *p* = 0.036)	N/A
- 50 days	−0.27 (95%CI, −0.33 to −0.20; *p* < 0.001)	−0.16 (95%CI, −0.23 to −0.09; *p* < 0.001)	N/A
Seropositive			
- 21 days	−0.03 (95% CI; −0.16 to 0.11; *p* = 0.680)	0.09 (95% CI; −0.05 to 0.23; *p* = 0.189)	−0.80 (95% CI; −0.84 to −0.74; *p* < 0.001)
- 50 days	−0.05 (95% CI; −0.18 to 0.09; *p* = 0.487)	0.12 (95% CI; −0.02 to 0.25; *p* = 0.096)	−0.88 (95% CI; −0.91 to −0.85; *p* < 0.001)

**Table 3:** Multivariate analysis (beta coefficient and 95% CI) of the ratio of increase from individual baseline total anti-SARS-CoV-2 RBD antibodies level and age, sex or baseline antibodies value in baseline seronegative (<0.8 U/L) and baseline seropositive (≥0.8 U/L) subjects receiving a complete cycle (i.e., two doses) of Pfizer COVID-19 mRNA BNT162b2 vaccine.

Antibody Status	Age	Sex (Males vs. Females)	Baseline Value
Seronegative			
- 21 days	-5 (95% CI; -7 to -3; *p* < 0.001)	-10 (95% CI; -66 to 45; *p* < 0.706)	N/A
- 50 days	-565 (95% CI; -1036 to -93; *p* < 0.001)	-51 (95% CI; -69 to -32; *p* = 0.019)	N/A
Seropositive			
- 21 days	0.4 (95% CI; -9.5 to 10.4; *p* = 0.930)	0.8 (95% CI; -274.4 to 276.0; *p* = 0.995)	-0.2 (95% CI; -0.4 to 0.0; *p* = 0.023)
- 50 days	-1.3 (95% CI; -13.8 to 11.3; *p* = 0.804)	19.1 (95% CI; -326.2 to 364.4; *p* = 0.913)	-0.3 (95% CI; -0.5 to 0.0; *p* = 0.022)

## Data Availability

Data supporting reported results are available upon request to the corresponding author.
